# Self-Powered Microfluidic System Based on Double-Layer Rotational Triboelectric Nanogenerator

**DOI:** 10.3390/mi16121386

**Published:** 2025-12-06

**Authors:** Yiming Zhong, Haofeng Li, Dongping Wu

**Affiliations:** State Key Laboratory of Integrated Chips and Systems, College of Integrated Circuits and Micro-Nano Electronics Innovation, Fudan University, Shanghai 200433, China

**Keywords:** triboelectric nanogenerator, microfluidic technology, self-powered system, droplet manipulation

## Abstract

Self-powered microfluidic systems represent a promising direction toward autonomous and portable lab-on-chip technologies, yet conventional electrowetting platforms remain constrained by bulky high-voltage supplies and intricate control circuitry. In this work, we design a triboelectric nanogenerator (TENG)-based microfluidic system that harvests mechanical energy for droplet manipulation without any external electronics. The TENG integrates two triboelectric units with a 25° phase offset, enabling periodic high-voltage generation. Finite element simulations elucidate the electric field distributions of the TENG and microfluidic chip, validating the operating principle of the integrated microfluidic system. Experimental studies further quantify the effects of electrode geometry and rotational speed on the critical drivable droplet volume, demonstrating stable transport over linear, S-shaped, and circular trajectories. Remarkably, the droplet motion direction can be instantaneously reversed by reversing the TENG rotation direction, achieving bidirectional control without auxiliary circuitry. This work establishes a voltage-optimized, structurally tunable, and fully self-powered platform, offering a new paradigm for portable digital microfluidics.

## 1. Introduction

Microfluidic technology, which enables precise manipulation of fluid volumes at the microliter to nanoliter scale, has become a popular method in biochemical analysis [1,2], environmental monitoring [3], drug synthesis [4,5], and high-throughput screening [6,7]. Droplet-based microfluidics, in particular, treats each droplet as an independent microreactor, facilitating programmable chemical reactions, parallelized diagnostics, and high-throughput analysis with minimal reagent consumption [8]. The rapid development of portable lab-on-chip and point-of-care testing devices has further underscored the need for compact, precise, and energy-efficient droplet actuation systems capable of autonomous operation [9,10]. Various droplet actuation strategies have been developed, including optical [11,12], thermal [13], acoustic [14], magnetic [15,16], and electric [17,18]. Among these, electrically controlled droplet technology has emerged as the most widely used technique due to its fast response, low power consumption, and ease of digital control [19]. Nevertheless, traditional droplet manipulation systems rely on external high-voltage sources (typically 60~1000 V) and complex electronic circuits to sustain actuation, which significantly increases system cost and design complexity [20]. This dependency limits their use in miniaturized, portable, and wearable devices.

The triboelectric nanogenerator (TENG), which converts mechanical motion into electricity through the combined effects of triboelectrification and electrostatic induction, offers an elegant solution to this problem [21,22,23,24]. Distinguished by their high output voltage, low current, light weight, and simple fabrication, TENGs provide an inherently safe and energy-autonomous power source for droplet manipulation [25]. Their ability to harvest energy from ubiquitous mechanical motions, including vibration, rotation, and human activity, makes them ideal for self-powered microfluidic applications [26,27,28]. In this regard, pioneering studies have explored the feasibility of TENG-driven droplet manipulation. Nie et al. demonstrated a freestanding-mode TENG driving a liquid-based micro vehicle, while its unidirectional motion mode and linear trajectory restricted its versatility [29]. Yu et al. later developed a rotational TENG with an electric brush commutation unit that enabled droplet splitting and merging; however, this architecture increased system complexity and required additional switching circuits to reverse the droplet movement direction [30]. More recently, Li et al. proposed a self-powered microfluidic system that drives droplets to move in a two-dimensional plane; however, the TENG electrodes were directly mapped one-to-one to those of the microfluidic chip, limiting the ability of long-range droplet transport [31]. These studies collectively indicate that current TENG-based microfluidic systems still suffer from limited flexibility and inadequate capability for scalable long-range droplet transport along complex trajectories without auxiliary circuit switching. Therefore, there is an urgent need for a TENG architecture that can independently generate stable periodic high-voltage signals while supporting versatile microfluidic path movement.

In this work, we present a self-powered microfluidic system that couples a double-layer rotational TENG with a PCB-based microfluidic chip. The TENG structurally combines two triboelectric generating units. Through rotational phase control, the TENG generates stable, periodic high-voltage outputs that can be flexibly mapped onto a designed microfluidic electrode array without additional mechanical parts. Based on this architecture, the working mechanism is systematically elucidated through COMSOL simulations, which reveal the evolution of electrode potentials under different rotational angles and clarify the electrostatic actuation process. Guided by these simulation results, microfluidic chips with electrode widths and gaps ranging from 0.5 mm to 1.5 mm are designed and fabricated, and experimental characterization further investigates the relationship between the critical drivable droplet volume and key parameters such as rotation speed, electrode width, and gap. Subsequently, stable droplet transport driven by manual operation was demonstrated on linear, S-shaped, and circular electrode pathways, and the droplet motion direction could be easily adjusted by simply changing the rotation direction of the TENG. Overall, this work delivers a voltage-optimized, architecture-flexible, and fully self-powered TENG-driven microfluidic platform that achieves long-range, scalable, and autonomous droplet actuation, offering a promising pathway toward next-generation energy-independent portable microfluidic systems.

## 2. Materials and Methods

### 2.1. Materials

The material for the electrodes was copper foil (thickness 60 μm), purchased from Xinye Technology Ltd., Suzhou, China. The fluorinated ethylene propylene (FEP, thickness 30 μm) and nylon (PA6, thickness 25 μm) were purchased from Wiltan Ltd., Shanghai, China. The polytetrafluoroethylene (PTFE, thickness 60 μm) was purchased from Qinxintiong Ltd., Changzhou, China. The sponges (EPE, thickness 3 mm, density 22 kg × m^−3^) were purchased from Suodu Ltd., Anqing, China. Deionized water with no surfactant, a resistivity of 18.25 MΩ⋅cm, a temperature of 25 °C, and a pH of 6.8 was employed to form droplets.

### 2.2. Fabrication of TENG

The TENG device consists of three main components: a top disk, a middle rotating disk, and a bottom disk. The top disk is an acrylic plate with an outer diameter of 240 mm and an inner diameter of 16 mm. A layer of copper foil was adhered to its lower surface and patterned into four electrodes, followed by the lamination of a nylon film. Similarly, the bottom disk (outer diameter: 230 mm; inner diameter: 16 mm) was coated with copper foil patterned into four electrodes and covered with the same nylon film. The middle disk is an acrylic plate with an outer diameter of 226 mm and an inner diameter of 8 mm. On both the upper and lower surfaces, a pair of fan-shaped sponges (thickness: 2 mm) was symmetrically attached, and each sponge layer was covered with a fluorinated ethylene propylene (FEP) film. During assembly, the top disk was first fixed to the upper surface of an acrylic cylinder (outer diameter: 240 mm, inner diameter: 230 mm, height: 50 mm). Subsequently, the middle rotor disk and bottom disk were inserted into the acrylic cylinder. It is noteworthy that the electrode array on the bottom disk was intentionally rotated by 25° relative to the top disk to introduce a phase shift between the two TENG units. Finally, an aluminum rod was inserted as the central shaft, which was connected to the stator through a bearing while being directly fixed to the rotor to transmit external torque.

### 2.3. Fabrication of Microfluidic Device

The microfluidic chip was fabricated on a printed circuit board (PCB) substrate pre-patterned with a specific electrode configuration. The electrode layout was first designed using commercial PCB design software (V2.240, JLCEDA, JLC Co., Ltd., Shenzhen, China) and manufactured by an industrial PCB supplier. PDMS (SYLGARD 184, Dow Corning Co., Ltd., Midland, MI, USA) was prepared by mixing the base polymer and curing agent at a volume ratio of 10:1. The mixture was stirred thoroughly and then degassed in a vacuum chamber to remove trapped air bubbles. Subsequently, the PCB substrate was placed on a spin coater, and a droplet of PDMS precursor was dispensed at the center. The PDMS was spin-coated at 500 rpm for 20 s to achieve uniform spreading, followed by 2000 rpm for 90 s. The coated substrate was then cured in a drying oven at 60 °C for 4 h, resulting PDMS dielectric layer had a thickness of 30 μm. After curing, a PTFE film was attached onto the PDMS surface.

### 2.4. Measurement and Characterization

The TENG was mechanically driven by a geared motor via a shaft coupler, and the rotational speed was adjusted using a speed controller. For open-circuit voltage measurements, the TENG output was connected to a high-voltage probe (HVP-40, Pintek Co., Ltd., Taiwan, China), which was then linked to a programmable electrometer (6514, Keithley Co., Ltd., Beaverton, OR, USA). The electrometer was connected to a data acquisition module (USB-6008, NI Co., Ltd., Austin, TX, USA), which recorded data through a LabVIEW-based program. For short-circuit current and transferred charge measurements, the TENG was directly connected to the electrometer without the high-voltage probe. The static contact angle was measured using the sessile-drop method (JY-82C, Dingsheng Co., Ltd., Chengde, China), and the corresponding roll-off angle was measured using the tilting-plate method (SDC-350, SinDin Co., Ltd., Dongguan, China). The indoor temperature and humidity were maintained at 25 °C and 40%RH during the measurements.

## 3. Results and Discussion

### 3.1. The Structure and Characteristics of TENG

**Figure 1:**
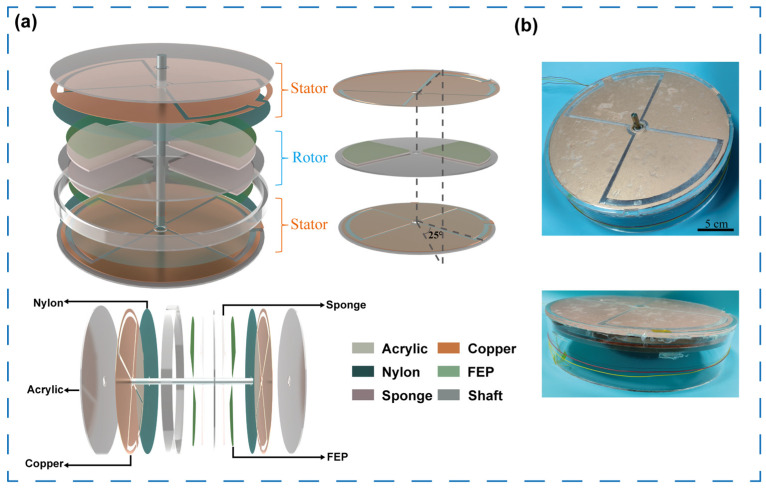
Diagram depicting the structure of the TENG. (**a**) 3D schematic diagram of the TENG, composed of a rotator and two stators. (**b**) Photographs of the TENG.

The rotational TENG is employed as the driving energy source for the self-powered microfluidic system. As illustrated in Figure 1a, the TENG consists of three coaxial circular components—a top disk, a middle disk, and a bottom disk. The top and bottom disks are fixed together with a cylindrical acrylic shell to form the stator, while the middle disk connected to a central-shaft acts as the rotor. Both the top and bottom disks are covered with copper electrodes and nylon films, whereas the surfaces of the middle disk are coated with FEP films adhered to sponges. Two independent triboelectric units are formed: one between the middle and lower plates, and the other between the middle and upper plates. As the rotor begins to rotate, charge transfer between the FEP and nylon surfaces induces alternating potentials on the copper electrodes. Importantly, the upper and lower electrode arrays are designed with a 25° phase offset, producing four distinct voltage outputs that vary periodically during rotation. The digital photograph is shown in Figure 1b.

**Figure 2:**
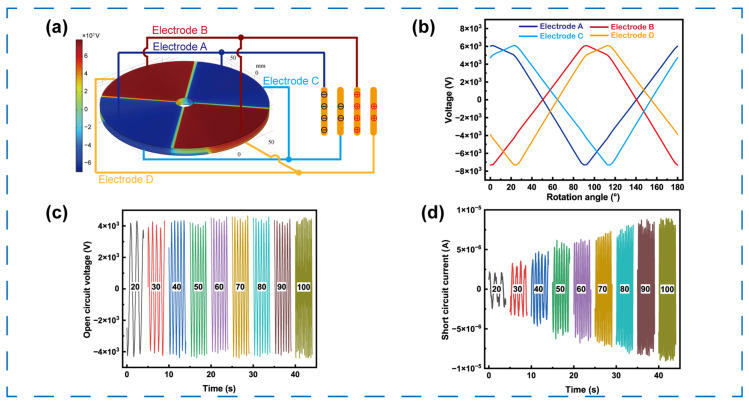
The output performance of the TENG. (**a**) Simulated electric potential distribution of the TENG. (**b**) Calculated open circuit voltage in a rotating circle. (**c**) The open circuit voltage and (**d**) the short circuit current of the TENG at different rotation speeds.

To validate the proposed structural design, electrostatic simulations were conducted using COMSOL Multiphysics (COMSOL Multiphysics 5.5). In this device, two opposite copper electrodes on the top disk were connected to form induced electrodes A and B, while those on the bottom disk were paired as electrodes C and D. The simulated potential distribution at the initial state is shown in Figure 2a. When the FEP film on the middle rotor is fully aligned with electrode A, the combined dielectric effects of FEP and nylon cause electrode A to exhibit the lowest potential (deep blue), whereas electrode B shows the highest potential (deep red). Consequently, positive and negative charges are induced on the surfaces of electrodes A and B, respectively. The potentials of electrodes C and D are relatively small in magnitude, appearing as light-blue and light-red regions, corresponding to smaller amounts of induced charge. To comprehensively analyze the potential evolution during operation, the middle disk was set to rotate counterclockwise from 0° to 180° in 2.5° increments, representing one full potential variation cycle due to the symmetry of the electrode configuration. The continuously simulated potential distributions at different rotational angles are provided in Figure S1 and Movie S1. The ground-referenced electrode potentials were extracted from the electrode backside as illustrated in Figure 2b. Initially, electrode A exhibited the highest voltage (~6000 V), and electrode B the lowest (~−7300 V), while electrodes C and D showed intermediate voltages of approximately +3000 V and −2300 V, respectively. With continuous rotation, the electrode voltages alternated periodically: when the rotation reached 90°, the polarities of A/B and C/D were completely reversed; when the rotation reached 180°, the open-circuit voltages returned to their initial values. This cyclic potential evolution agrees well with the theoretical prediction and confirms that the designed TENG can deliver a stable, periodically varying high output voltage suitable for droplet manipulation.

Subsequently, the fabricated TENG was connected to a geared motor to evaluate its actual electrical output performance. By adjusting the motor speed through a controller, the output characteristics at different rotation speeds were obtained. As shown in Figure 2c, when the rotation speed increased from 20 rpm to 100 rpm, the open-circuit voltage remained nearly constant at approximately 4300 V. Meanwhile, the short-circuit current increased from 2 μA to 9 μA, while the transferred charge remained approximately 930 nC, as illustrated in Figure 2d and Figure S2a. It should be noted that the absolute voltage amplitude in simulation is higher than the experimentally measured open-circuit voltage. This discrepancy is mainly attributed to practical non-idealities that are not included in the ideal electrostatic model. In experiments, residual moisture and humidity accelerate charge dissipation, while surface roughness reduces the true contact area and thus lower charge-transfer efficiency. In addition, limited load uniformity during rotation, together with assembly-induced mechanical stresses can lead to non-uniform contact conditions. Collectively, these effects lower the measured voltage amplitude while preserving the same periodic output trend.

Furthermore, the open-circuit voltage, short-circuit current, and transferred charge of TENG as humidity increases from 20% to 60% are compared, as shown in Figure S2b. The durability of the device was also a crucial parameter for assessing its output stability. In this study, nylon films were laminated over the copper electrodes to prevent direct contact between the metal electrodes and dielectric films, effectively reducing friction and mechanical abrasion. As shown in Figure S3, the device was subjected to durability tests of 500, 1000, 5000, and 10,000 operation cycles, and the output remained largely stable throughout the cycling. After 10,000 cycles, the peak open-circuit voltage exhibited only a slight decrease of 6.5%, remaining at approximately 4000 V. Photographs taken after 10,000 cycles (Figure S4) show no obvious macroscopic wear or delamination of the FEP film, supporting the mechanical stability of the TENG.

### 3.2. Working Principle of Self-Powered Microfluidic System

**Figure 3:**
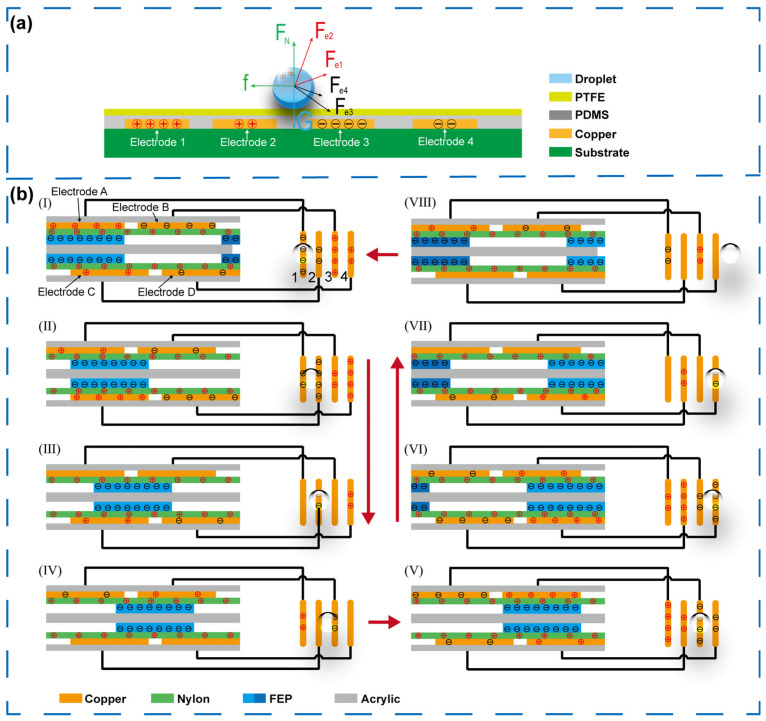
(**a**) Structure of the microfluidic chip. (**b**) Schematic working principle of the self-powered microfluidic system.

The microfluidic chip serves as an important component of the microfluidic system, enabling droplet manipulation on its surface through a controllable electric field. As illustrated in Figure 3a, the chip mainly consists of four layers: a substrate, patterned driving electrodes, a polydimethylsiloxane (PDMS) dielectric layer, and a polytetrafluoroethylene (PTFE) hydrophobic surface layer. The PDMS layer is required to suppress discharge and air breakdown under the small electrode gap, thereby ensuring stable droplet transport; meanwhile, its thickness is controlled to avoid excessive attenuation of the effective actuation field. The PTFE film provides a hydrophobic interface to facilitate smooth droplet motion, with a static contact angle of 140° and a roll-off angle of 20° (Figure S5). To analyze the motion mechanism, a simplified force model was established based on the electrostatic potential distribution when the TENG rotor is positioned at a rotation angle of 90°, as shown in Figure S6. When exposed to air, the droplet naturally acquires a small positive charge through charge exchange with atmosphere [32]. The surface of electrodes 1–4 carries varying amounts of positive and negative charge, exerting multiple forces on the droplet, including gravitational force (G), normal support force (*F*_N_), frictional resistance (*f*), and electrostatic forces (*F*_e1,_ *F*_e2,_ *F*_e3,_ *F*_e4_) from each electrode. When the droplet is located between electrodes 2 and 3, the electrostatic forces from electrodes 1 and 2 counteract gravity in the y-direction while repelling the droplet in the x-direction, driving it rightward. Simultaneously, the forces from electrodes 3 and 4 enhance friction but attract the droplet rightward along the *x*-axis. As long as the resultant horizontal force (*F*_x_) exceeds frictional resistance (*f*), the droplet continues to move to the right. When the droplet reaches electrode 3, the electrostatic attraction from electrodes 1 and 2 diminishes, while that from electrodes 3 and 4 increases, causing a temporary reduction in velocity or even stop. If the droplet still retains kinetic energy sufficient to cross the boundary of electrode 3, the direction of *F*_e3_ reverses and dominates, pulling the droplet back toward electrode 3. Therefore, the charge magnitude and polarity on electrodes 1–4 must vary periodically to ensure continuous, directional droplet motion.

When a microfluidic chip is integrated with the TENG, the detailed working mechanism of the complete microfluidic system is illustrated in Figure 3b. The rotor (middle disk) and stator (top and bottom disks) of the TENG are covered with FEP and nylon possessing different triboelectric polarities. Upon contact, these films exchange charges and acquire equal amounts of opposite polarity. For a given net transferred charge, because the total FEP area is half that of nylon, the surface charge density on the rotor is approximately twice that on the stator. Under the combined influence of the triboelectric charges, the copper electrodes on both disks become electrostatically induced, and the amount of induced charge depends on the rotor’s angular position. In this system, the induced electrodes A, C, B, and D of the TENG are connected to the corresponding control electrodes 1, 2, 3, and 4 on the microfluidic chip. When induced charges appear on the TENG electrodes, opposite charges of equal magnitude are generated on the microfluidic control electrodes, forming an electric field that drives droplet movement. In the initial state I, the upper FEP film fully overlaps with the upper-left copper electrode A, which induces abundant positive charge on electrode A and corresponding negative charge on the connected control electrode 1 of the microfluidic chip. Simultaneously, electrode B accumulates negative charges, inducing positive charges on control electrode 3. Because the lower disk is offset by 25°, its FEP layer only partially overlaps with electrodes C and D, which therefore carry moderate positive and negative charges, leading to moderate negative and positive charges on control electrodes 2 and 4, respectively. Under these conditions, the droplet remains stably above electrode 1. As the rotor turns to state II, the overlap between the upper FEP layer and copper electrodes decreases, resulting in moderate positive and negative charges on electrodes A and B, while the lower FEP film fully covers electrodes C and D, producing abundant negative and positive charges. The droplet is consequently attracted toward electrode 2 under the newly formed electric-field gradient. When rotation continues to state III, the overlap areas between the upper FEP layer and electrodes A and B become equal, neutralizing their surface charges, whereas the asymmetric overlap on the lower disk produces moderate positive and negative charges on electrodes C and D, inducing opposite charges on electrodes 2 and 4, which stabilizes the droplet above electrode 2. At state IV, electrodes A and B regain moderate positive and negative charges while the lower electrodes C and D become neutral due to symmetrical overlap, generating a directional field that drives the droplet toward electrode 3. As rotation continues to state V, the upper FEP film fully overlaps with electrode B, reversing the polarity of the earlier configuration and causing the droplet to move onto electrode 3. In subsequent state VI to state VIII, the periodic overlap and separation between the FEP films and copper electrodes continuously alternate the induced charges across electrodes A–D, forming a dynamic sequence of attraction and repulsion forces that guide the droplet from electrode 3 to electrode 4 and beyond. After a 180° rotation, the charge configuration of all electrodes returns to that of state I, completing one full cycle. Continuous rotation of the TENG thus produces a periodic and phase-shifted potential distribution that enables sustained, autonomous, and directional droplet motion.

### 3.3. Construction of Microfluidic Chip

**Figure 4:**
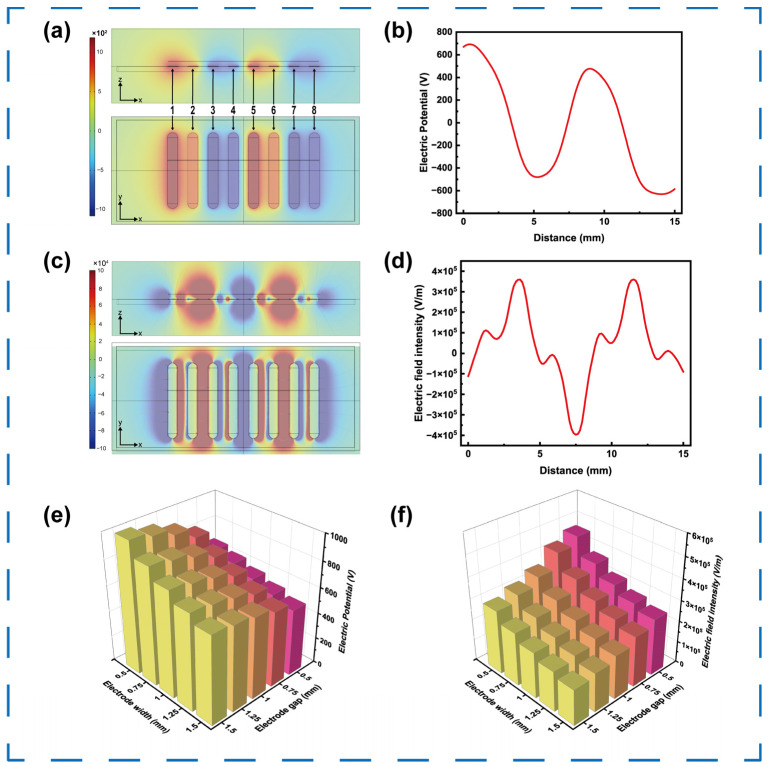
Simulation of microfluidic chip. (**a**) The simulated distribution and (**b**) the extracted data of electric potential. (**c**) The simulated distribution and (**d**) the extracted data of electric field intensity. (**e**) The maximum electric potential and (**f**) the maximum electric field intensity versus the electrode width and gap.

According to the previous analysis, the droplet actuation in this self-powered microfluidic system primarily depends on the electrostatic force generated by the potential distribution across the controlled electrode array. To elucidate the electric field conditions, COMSOL Multiphysics simulations were performed on the designed microfluidic chip. As illustrated in Figure 4a, electrodes 1, 2, 5, and 6 exhibit positive potentials, while electrodes 3, 4, 7, and 8 are negatively potentials. Figure 4b shows the potential values extracted 1 mm above the electrode surface, where electrodes 1, 2, 5, and 6 reach a maximum potential of approximately +700 V, while electrodes 3, 4, 7, and 8 display minimum potentials around −630 V. The corresponding x-direction electric field component (*E*_x_) is depicted in Figure 4c, which directly determines the droplet motion. Figure 4d reveals the extracted field distribution 1 mm above the electrode plane. The strongest positive *E*_x_ of 3.59 × 10^5^ V/m is between electrodes 2 and 3, and similarly between electrodes 6 and 7. The strongest negative *E*_x_ of −3.96 × 10^5^ V/m appears between electrodes 5 and 6. Consequently, if a droplet is placed between electrodes 2 and 3, it will move by a rightward force. As the droplet passes on electrode 3, the decreasing field will decelerate its motion. If the droplet slightly moved across electrode 3, the reversed field pulls it back, consistent with the theoretical electrostatic model described above.

Under a fixed number of surface charges, the geometry and layout of control electrodes significantly affect the electric field distribution and hence the droplet manipulation capability. To investigate this effect, the electrode length was fixed at 10 mm, as this value is sufficiently long for the droplet to interact primarily with the central electrode region, thereby minimizing fringing effects and enabling stable motion. The dependence of the peak surface potential and electric-field intensity on electrode length is shown in Figure S7. We then varied the electrode width and gap from 0.5 mm to 1.5 mm in 0.25 mm increments, resulting in 25 electrode combinations. As shown in Figure 4e, the peak surface potential decreases sharply with increasing electrode width, primarily because a wider electrode reduces the surface charge density. Conversely, as the gap increases, the peak potential gradually rises due to the weaker electrostatic coupling from the adjacent oppositely charged electrode to the peak-potential region. As depicted in Figure 4f, the peak electric field intensity *E*_x_ decreases consistently with both increasing electrode width and gap, since *E*_x_ is influenced by both the potential difference and the separation distance; a lower potential and larger gap together result in a weaker effective driving field.

**Figure 5:**
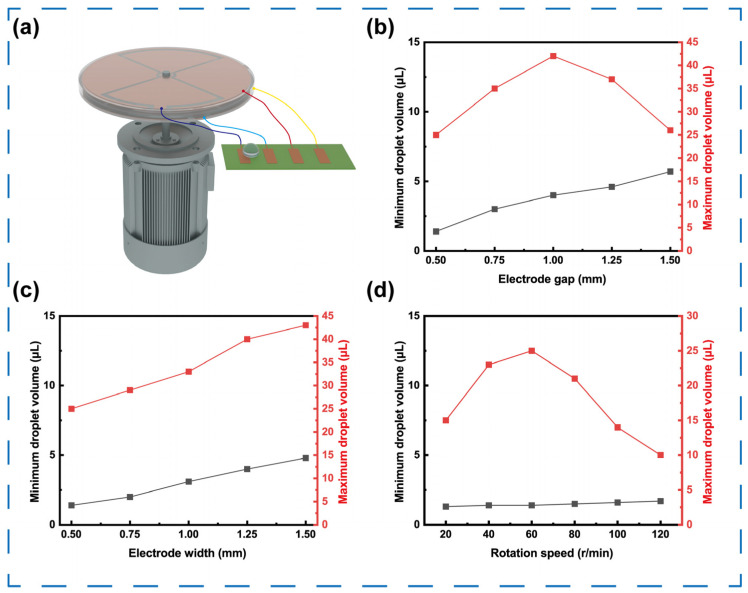
Performance of the microfluidic system for driving the critical volume of droplets. (**a**) schematic of the microfluidic system driven by a geared motor. Maximum (red) and minimum (black) critical volumes of a droplet versus the (**b**) electrode gap, (**c**) electrode width, and (**d**) rotation speed.

To experimentally verify these effects, microfluidic chips with different electrode geometries were fabricated and tested under a constant TENG rotation speed of 60 rpm (Figure 5a). For chips with a fixed electrode width of 0.5 mm and varying gaps (0.5–1.5 mm), the minimum drivable droplet volume gradually increased from 0.3 μL to 4 μL as the gap widened (Figure 5b). This occurs because smaller droplets carry less charge and thus require stronger electric fields to overcome surface friction, whereas *E*_x_ weakens with increasing gap. Meanwhile, the maximum drivable droplet volume first increased and then decreased, reaching an optimum of 26 μL at a 1.0 mm gap before dropping to 15 μL at 1.25 mm. When the gap is too small, larger droplets overlap multiple electrodes simultaneously and experience opposing forces, hindering motion; when gap is too large, weakened electric fields and higher viscous drag restrict droplet transport. For chips with a fixed gap of 0.5 mm and varying electrode widths (0.5–1.5 mm, Figure 5c), both the minimum and maximum drivable volumes increased from 0.3 μL to 3 μL and from 3 μL to 22 μL, respectively. A wider electrode requires a larger droplet to span multiple electrodes for continuous motion, whereas smaller droplets fail to cross the electrode midpoint before polarity reversal, resulting in motion cessation.

The influence of TENG rotational speed on droplet manipulation was further evaluated using microfluidic chips with an electrode width and gap of both 0.5 mm (Figure 5d). As the rotational speed increased from 20 to 60 rpm, the maximum drivable droplet volume increased from 25 μL to 30 μL, while further acceleration above 80 rpm caused a sharp decline to 22 μL. At low speeds, the field switching period is sufficiently long for the droplet to accelerate and cross the electrode midpoint before polarity reversal, which facilitates the transport of larger droplets. However, at high speeds, rapid field switching reverses polarity before the droplet reaches the next electrode, converting driving force into resistance and causing oscillation rather than directional motion. In contrast, the minimum drivable volume remained nearly constant since smaller droplets possess lower friction and higher velocity, allowing them to keep pace with the changing electric field even at 120 rpm.

### 3.4. Demonstration

**Figure 6:**
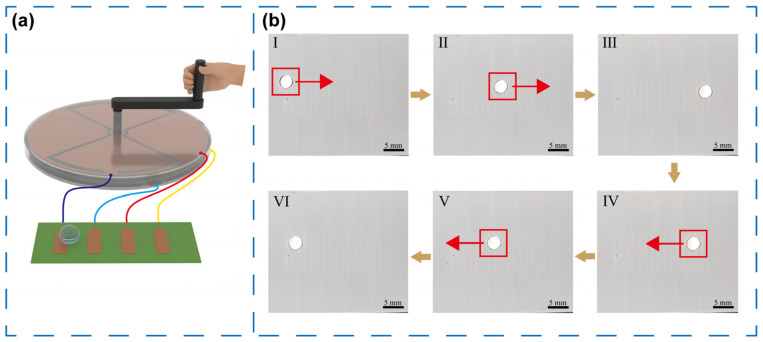
(**a**) Diagram of the self-powered microfluidic system with a straight line path. (**b**) Droplet’s motion states in a straight line at different time points, move forward (I–III) and backward (IV–VI).

Microfluidic systems enable precise manipulation of minute liquid volumes, where each droplet functions as an independent reaction unit, offering enhanced accuracy, accelerated reaction kinetics, and minimal sample consumption. However, conventional systems typically rely on complex control circuitry and external power supplies, limiting their usability in remote or resource-constrained environments. To overcome this challenge, a hand-crank module was mounted on the central shaft of the TENG, as illustrated in Figure 6a, enabling mechanical energy from manual rotation to be directly converted into electrical output for driving the microfluidic chip. As shown in Figure 6b, a 10 μL deionized water droplet was deposited onto the microfluidic chip surface (electrode width: 0.5 mm, electrode gap: 1 mm) and actuated through hand-driven TENG rotation. During clockwise rotation (states I–III), the droplet moved steadily to the right under the alternating electrostatic field, demonstrating precise and stable transport. When the rotation direction was reversed (stages IV–VI), the droplet correspondingly shifted leftward, highlighting an advantage that it enables bidirectional motion control simply by altering the TENG’s rotation direction. The full motion process, including forward and reverse transport at two distinct speeds, is provided in Movie S2, confirming the system’s controllability and dynamic adaptability. To characterize the electrostatic properties of the working droplet, we measured the net charge carried by droplets of different volumes (Figure S8a). Moreover, the droplet electrode-crossing time extracted at different rotational speeds is presented in Figure S8b to quantify the actuation dynamics. In addition, high-speed imaging snapshots at different time points during droplet transport are provided in Figure S9.

**Figure 7:**
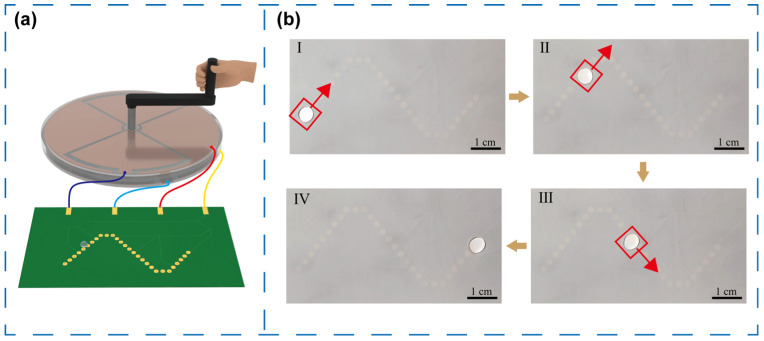
(**a**) Diagram of the self-powered microfluidic system with S-shape path. (**b**) Droplet’s motion states in an S-shaped trajectory at different time points.

In many practical microfluidic applications, droplets are required to follow non-linear trajectories. To demonstrate this capability, a microfluidic chip with an S-shaped path was designed, consisting of multiple octagon electrodes (outer circle diameter: 2 mm) with an interelectrode gap of 2 mm (Figure 7a). As shown in Figure 7b, a 50 μL droplet was driven to follow the S-shaped path continuously, reaching the endpoint within 19 s at an average velocity of approximately 4 mm/s. The motion remained stable throughout, and reversing the TENG rotation direction instantly reversed the droplet motion, as recorded in Movie S3.

**Figure 8:**
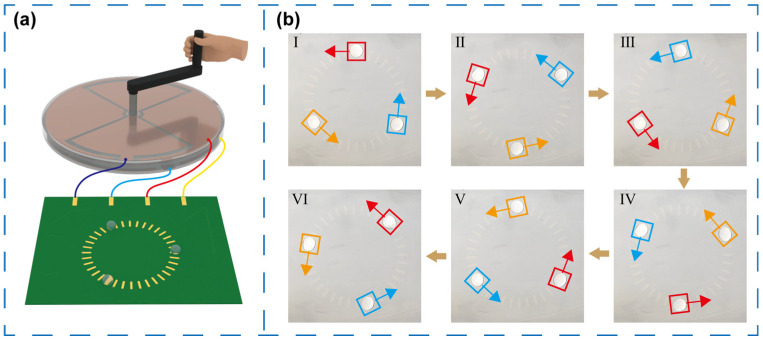
(**a**) Diagram of the self-powered microfluidic system with circular path. (**b**) Motion states of three droplets in a circle at different time points.

To further demonstrate sustained and synchronized driving capability of the self-powered microfluidic system, a circular path microfluidic chip consisting of 36 electrodes was designed and fabricated. Each electrode was a rounded rectangle with a width of 2 mm and a length of 5 mm, arranged at an angular interval of 10°, forming an annulus with an inner diameter of 40 mm and an outer diameter of 50 mm. As illustrated in Figure 8a, the chip was connected to the TENG to establish a self-powered microfluidic system, and three droplets of deionized water (100 μL each) were dispensed at different positions along the circular path. The picture in Figure 8b shows that all droplets were simultaneously driven in a clockwise direction while maintaining a nearly constant gap throughout the motion. Moreover, as recorded in Movie S4, under both low and high rotation speeds of the TENG, the three droplets were be stably propelled along a circular trajectory, and the motion direction can be conveniently reversed by changing the rotation direction of the TENG. These results confirm that the developed system achieves reliable, long-term droplet circular movement with fully reversible directional control.

To further clarify the novelty and advantages of our device, we added a comprehensive comparison with previously reported TENG-based microfluidic systems. As summarized in Table 1, our system uniquely combines advantages of multiple motion paths, ease of human operation, operation without additional switching circuits, continuous droplet movement, and long-distance transport, highlighting its potential of the proposed architecture in self-powered microfluidic platforms.

## 4. Conclusions

In summary, a self-powered microfluidic system that couples a double-layer rotational TENG with a PCB-based microfluidic chip was successfully developed, enabling long-range and reversible droplet manipulation. The unique phase-shifted configuration of two triboelectric units provides stable, periodic high-voltage outputs without any auxiliary circuitry. Finite element simulations elucidated the dynamic evolution of potential distribution across four induced electrodes and the microfluidic driving electrodes, revealing the mechanism principle for droplet manipulation. Systematic experimental analysis further identified the influence of electrode geometry and rotational speed on the effective driving field and the critical droplet volume for stable motion. Functionally, the platform achieves manually operated, bidirectional transport on linear tracks; continuous guidance of a 50 μL droplet along an S-shaped path; and synchronized circulation of three 100 μL droplets on a circular path. Notably, the droplet movement direction can be instantly switched by reversing the TENG rotation, achieving bidirectional control without auxiliary circuitry, a significant advancement beyond conventional TENG-based microfluidic systems. By directly harvesting low-frequency mechanical inputs into a periodically varying electrostatic field, the platform removes high-voltage supplies and control circuits, providing an energy autonomous, flexible microfluidic system for portable lab-on-chip, biochemical analysis, and point-of-care diagnostics.

## Figures and Tables

**Table 1:** The comparison with other reported TENG-based microfluidic system.

Mode of TENG	Output of TENG	Size of TENG	Multiple Motion Paths	Ease for Human Operation	Without Additional Switching Circuits	Continuous Movement	Long Distance Movement	Volume of Droplets	Ref.
Sliding freestanding	3000 V	100 mm × 350 mm	No	Yes	No	No	No	70 nL~40 μL	[29]
Rotary freestanding	1500~5000 V	Diameter: 80 mm	Yes	Yes	No	Yes	Yes	1 μL~500 μL	[30]
Sliding freestanding	35,000 V	150 mm × 100 mm	No	Yes	No	Yes	No	3 μL~40 μL	[33]
Rotary freestanding	730~5260 V	Diameter: 30 mm	No	No	Yes	No	No	-	[34]
Rotary freestanding	75~180 V	-	Yes	No	No	Yes	Yes	1.6 μL~2.4 μL	[35]
Sliding freestanding	1610 V	Each unit: 50 mm × 50 mm	Yes	Yes	Yes	Yes	No	20 nL~1.4 mL	[31]
Rotary freestanding	~4300 V	Diameter: 240 mm	Yes	Yes	Yes	Yes	Yes	0.3 μL~43 μL	This work

## Data Availability

The original contributions presented in this study are included in the article/Supplementary Material. Further inquiries can be directed to the corresponding author.

## Supplementary Materials

The following supporting information can be downloaded at: https://www.mdpi.com/article/10.3390/mi16121386/s1. Figure S1: Simulated electric potential distributions under different rotation angle; Figure S2: Output performance of the TENG under different humidity; Figure S3: Durability test of the TENG; Figure S4: Contact angle of PTFE films; Figure S5: Photograph of FEP films; Figure S6: Force analysis of a droplet; Figure S7: The simulation results of varying electrode length; Figure S8: The charge and movement of droplets; Figure S9: Microfluidic movement process; Movie S1: Dynamic simulated electric potential distributions; Movie S2: Manipulate droplet in a straight path; Movie S3: Manipulate droplet in a S-shaped path; Movie S4: Manipulate droplet in a circular path; Table S1. The parameters used in the simulation model of TENG.
