# Supplementary material for: Self-Powered Microfluidic System Based on Double-Layer Rotational Triboelectric Nanogenerator
Source: Micromachines (Basel). 2025 Dec 6;16(12):1386. doi: 10.3390/mi16121386 (PMC12735088; doi:10.3390/mi16121386)
Supplement: Supplementary file 1 [file micromachines-16-01386-s001.zip › Supplementary Material.pdf]

Supplementary Material

Self-Powered Microfluidic System Based on Double-Layer Rotational Triboelectric Nanogenerator

Yiming Zhong, Haofeng Li and Dongping Wu *

State Key Laboratory of Integrated Chips and Systems, College of Integrated Circuits and Micro-Nano Electronics Innovation, Fudan University, Shanghai 200433, China

* Correspondence: dongpingwu@fudan.edu.cn

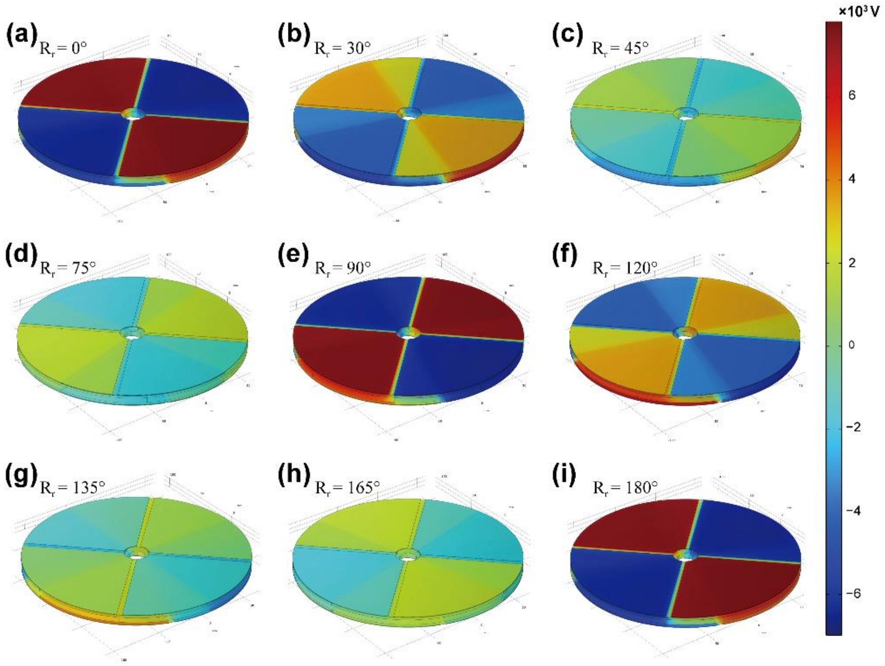

Figure S1. Simulated electric potential distributions under different rotation angle: (a) 0°, (b) 30°, (c) 45°, (d) 75°, (e) 90°, (f) 120°, (g) 135°, (h) 165°, (i) 180.

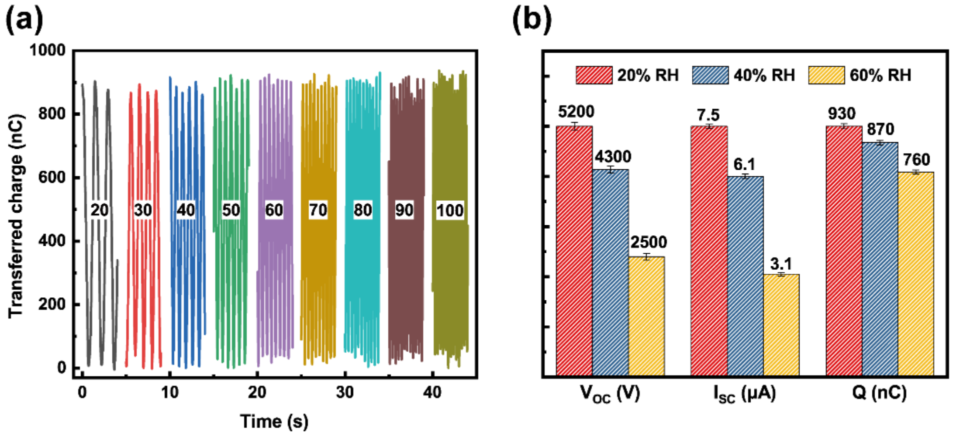

Figure S2. (a) The transferred charge of the TENG at different rotation speeds. (b) The output performance of the TENG under different humidity.

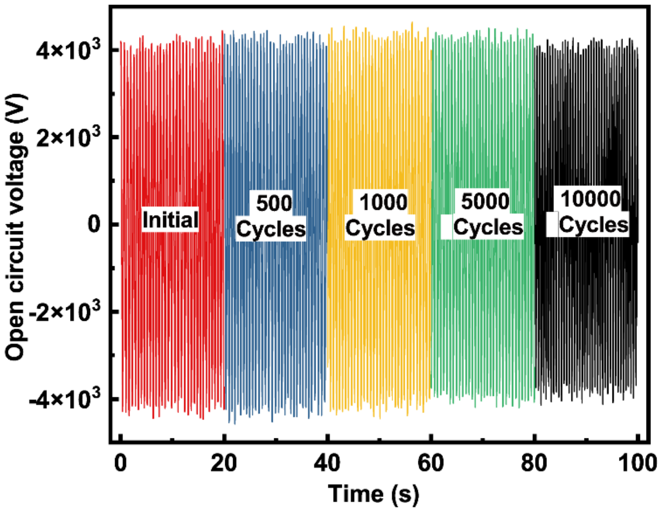

Figure S3. Durability test of the TENG

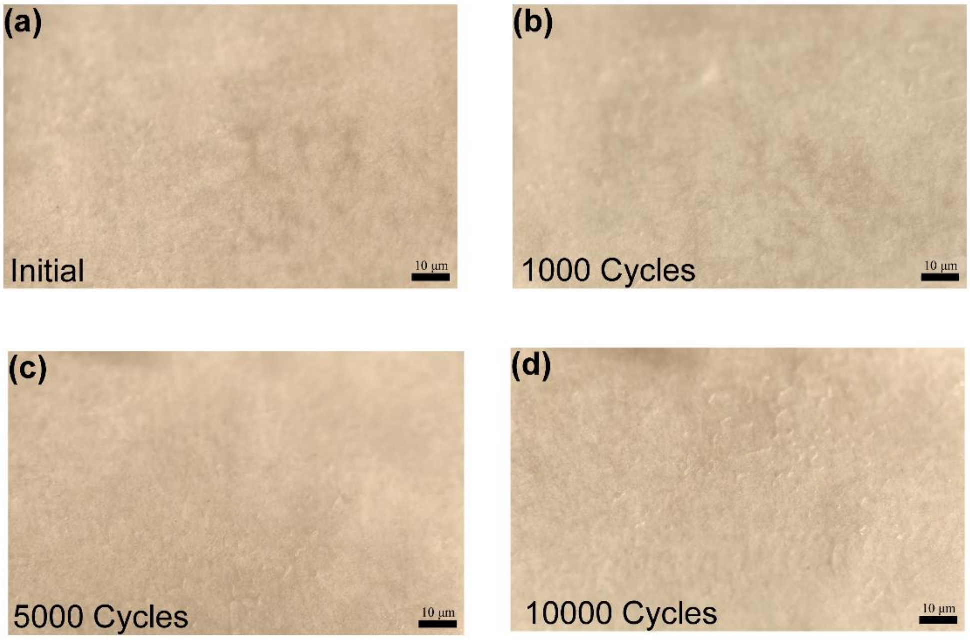

Figure S4. photographs of the FEP film after 1000, 5000, and 10000 operation cycles

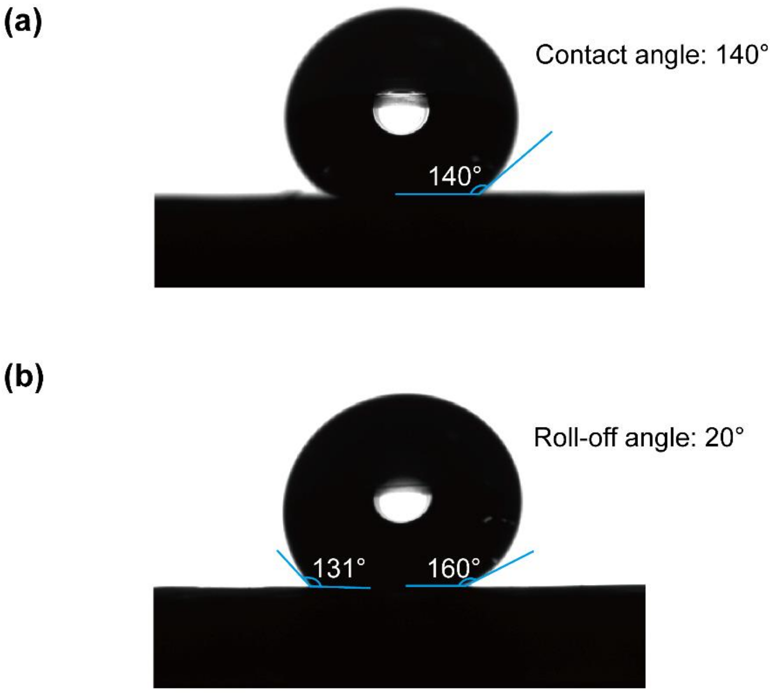

Figure S5. (a) The static contact angle and (b) roll-off angle of the PTFE film.

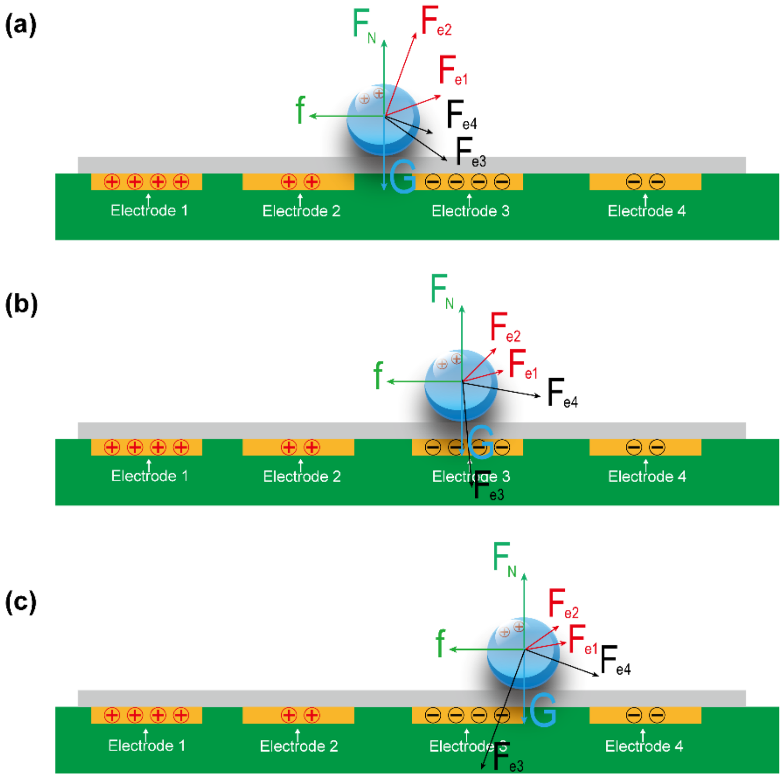

Figure S6. Force analysis of a droplet at different positions: (a) between electrode 2 and electrode 3, (b) above electrode 3, and (c) beyond electrode 3.

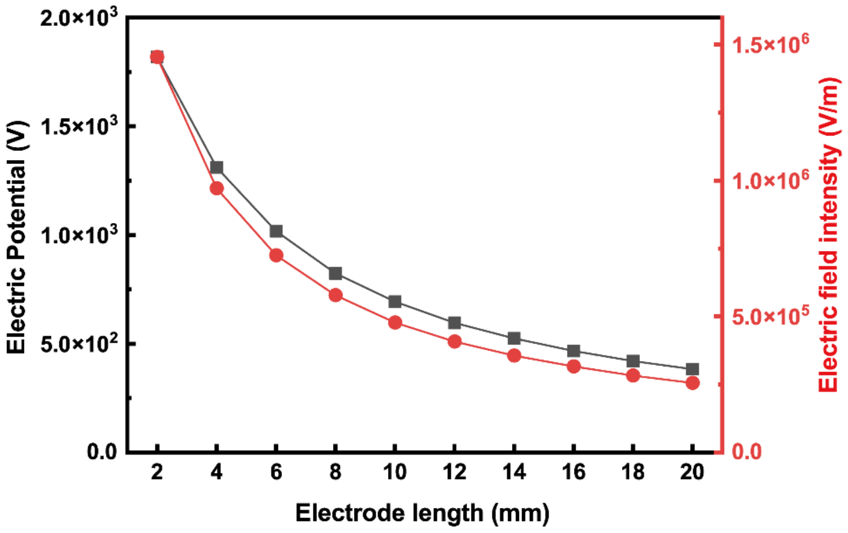

Figure S7. The maximum electric potential and electric field intensity at different electrode lengths.

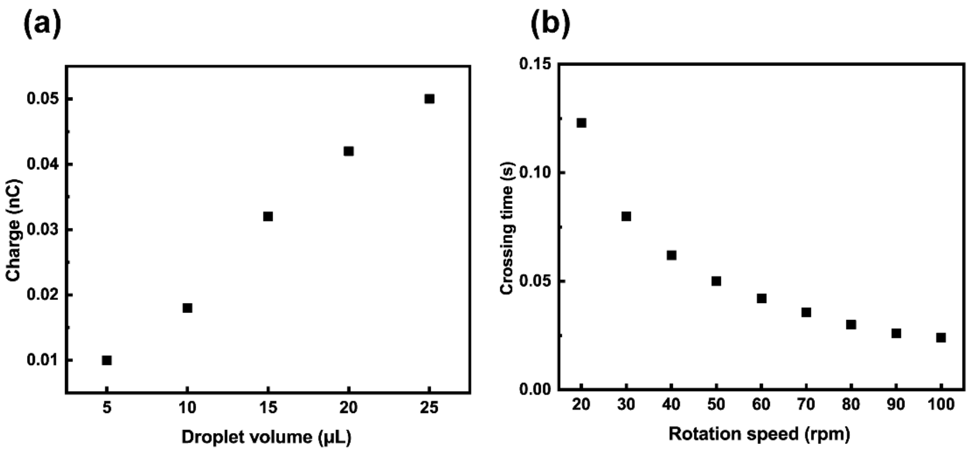

Figure S8. (a) The electric charge carried by droplets of different volumes. (b) The electrode-crossing times at different rotational speeds.

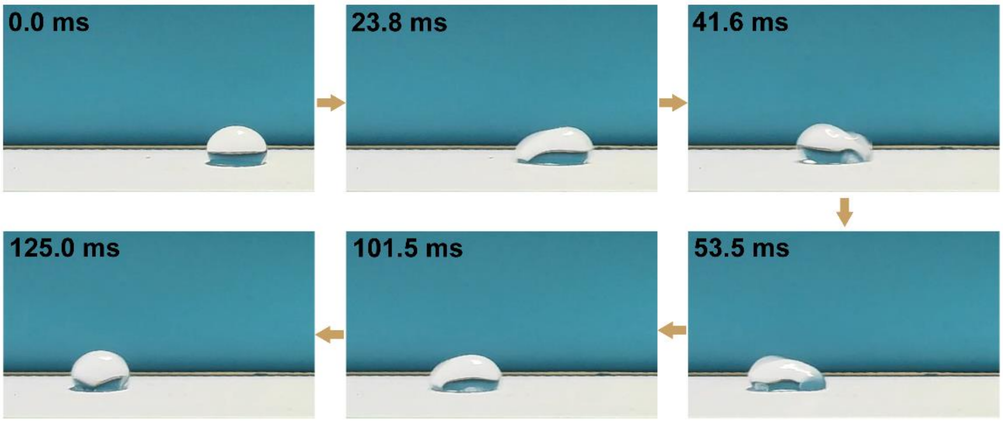

Figure S9. Sequential snapshots of a 10 μL droplet in the microfluidic movement process.

Table S1. The parameters used in the simulation model of TENG.

parameters	**nylon**	**FEP**	**Copper**	**Air**	**Sponges**	**PDMS**
Relative permittivity	2.1	2.1	1	1	4	2.7
Electrical conductivity (S/m)	5×10^{-15}	1×10^{-17}	5.81×10^{7}	5×10^{-16}	5×10^{-15}	1×10^{-15}
Surface charge density (C/m^2)	1.2×10^{-5}	-2.6×10^{-5}	-	-	-	-
Mesh	User-controlled/General physics/Extra fine					
Geometrical Boundary	Infinite element domain					
Boundary condition	Charge conservation/Zero charge/Initial values/Ground/Surface charge density/Floating potential					
Study	Stationary					
Physics	Electrostatics					
Rotation definition	Parametric sweep					
Distance between FEP and nylon	0.1 mm					
